# Is Covid-19 community level testing effective in reaching at-risk populations? Evidence from spatial analysis of New Orleans patient data at walk-up sites

**DOI:** 10.1186/s12889-021-10717-9

**Published:** 2021-04-01

**Authors:** Julie H. Hernandez, Dimitris Karletsos, Jennifer Avegno, Chantell H. Reed

**Affiliations:** 1 Department of Health Policy and Management, Tulane University School of Public health and Tropical Medicine, New Orleans, LA USA; 2 New Orleans Health Department, New Orleans, LA USA

**Keywords:** Covid-19, Coverage, Accessibility, New Orleans, Vulnerable populations, GIS

## Abstract

**Background:**

This paper evaluates the increase in coverage and use of Covid-19 testing services for vulnerable and hard-to-reach populations through the introduction of community-based walk-up sites in New Orleans, LA. While most GIS work on Covid-19 testing coverage and access has used census tract or ZIP code aggregated data, this manuscript is unique in that it uses individual level demographics and exact addresses to calculate distances actually traveled by patients.

**Methods:**

We used testing data recorded for 9721 patients at 20 sites operating in May–June 2020. The dataset includes detailed age, race and ethnicity, and testing results as well as the exact address of each individual. Using GIS, we estimated changes in testing coverage for minority neighborhoods and calculated the actual distance covered by individuals. Logistic regression and multivariate linear regression were used to identify socio-demographic variables associated with distance travelled to and used of nearest testing site. We used a secondary dataset from drive-through sites to evaluate change in coverage at the census tract level for the metropolitan area.

**Results:**

Walk-up sites significantly increased testing availability in New Orleans, and specifically in minority neighborhoods. Both African Americans and Asians were more likely (14.7 and 53.0%) to be tested at the nearest walk-up site. They also covered shorter distances to get tested. Being elderly was also significantly and positively associated with testing at the nearest site. Hispanics, however, were not associated with increased proximity to and use of nearest sites, and they traveled an additional 0.745 km to get tested. Individuals who tested positive also travelled significantly longer distances to obtain a test.

**Conclusions:**

Walk-up sites increased testing availability for some vulnerable populations who took advantage of the sites’ proximity, although inequalities appear at the metropolitan scale. As cities are planning community vaccination campaigns, mobile, walk-up sites appear to improve both coverage and accessibility for hard-to-reach populations. With adequate technical (vaccine dose refrigeration) and messaging (addressing reticence to immunization) adaptations, they could constitute a key complementary approach to health facility points of delivery.

## Introduction

In the months following the Covid-19 outbreak in the United States, hospitalization and mortality data clearly indicated that racial and ethnic minorities and socio-economically deprived areas were bearing a higher burden of disease and death than other groups [1–4]. Long-standing socio-economic and health disparities among African Americans, Hispanics, Native Americans and Alaska Natives compounded higher exposure to the virus, limited access to care, and higher risks of developing more severe disease [5]. Furthermore, the evidence supporting these disparate outcomes was partially myopic because testing data was often incomplete, despite calls from scientists and international health officials to systematically include race and ethnicity information in testing forms [6, 7], or biased due to the location of testing facilities. Early evidence from New York City indicated that people living in working-class and minority neighborhoods were less likely to get tested than residents of white and wealthier areas [8], and similar concerns were raised regarding testing disparities between rural and urban dwellers in Florida [9], and among ethnic minorities in Seattle [4]. As most tests were performed at health facilities, this partially reflected well-established disparities in access to formal care for vulnerable populations [10]. This created dangerous blind spot in testing coverage, which blurred the true burden of disease and hindered early cluster detection, contact tracing and follow-up care in vulnerable communities.

In April 2020, Orleans Parish, LA had the 4th highest number of Covid-19 death per capita in the country [11]. The county of 350,000 residents overlaps the city of New Orleans’ boundaries, with the surrounding parishes of Jefferson and St Bernard housing most of its suburban population. Orleans parish is a predominantly minority county, with 23.7% of its population living under the poverty line, and high levels of chronic illness and comorbidities [12]. At that time, three drive-through testing sites operated with support from the Federal Government and the National Guard. Initially reserved to symptomatic people, those sites required patients to drive or be driven to the site in order to be tested from their car for better efficiency and safety. This drive-through model however raised two associated concerns: almost one in five individuals living in New Orleans do not own a private vehicle (18.5% of Orleans Parish residents per 2019 ACS 5-Year Estimates) and minorities, particularly African Americans – already suspected to be disproportionately affected by severe forms of Covid-19 – were also the least likely to own a private vehicle.

To address this situation, the New Orleans Health Department (NOHD) partnered with a local healthcare network (LCMC) to deploy mobile testing sites throughout the city. The sites’ locations were selected to increase coverage at the city level with the specific objective to improve testing services in low-income and minority neighborhoods. Upcoming sites were advertised in the media and at the neighborhood level and patients would visit them on foot, register their information and get tested by LCMC personnel. All procedures were entirely free of charge and people were not required to present an ID. Interpreters and flyer in Spanish and Vietnamese were available on most sites. This design was meant to improve local awareness of testing service availability, offer shorter travel time, eliminate the barrier of cost, and improve local residents’ chances to be early in line, overall increasing likelihood of use.

Mobile sites were operating on a rotating basis, each opened 2 to 3 days before moving to the next community. Thus, theses sites were available at different locations at different points in time, with only one or two sites at most being open at the same time over the period considered. As NOHD primarily manages health issues and programs affecting the residents of the city of New Orleans, whose boundaries are coterminous with Orleans Parish, all walk-up sites were located within that parish, although access was not restricted for residents of other parishes (whose health programs are under the responsibility of the State of Louisiana).

The installation of these mobile sites and the collection of detailed demographic data and exact address of each person tested provided a unique opportunity to investigate the distances actually travelled by testing services users. Whereas most testing coverage analyses conducted to date have used potential accessibility indicators calculated with socio-demographic data aggregated at the census tract or ZIP code level [8, 13, 14], the dataset presented here circumvents the risks of ecological fallacy and provides individual level variables that will allow us to test two hypotheses [1]: whether walk-up community sites increased testing services availability for the vulnerable populations they were intended to reach, and [2] whether the distance travelled to get tested decreased for certain racial, ethnic and age groups.

The objective of this paper is to assess whether walk-up community sites increased access, understood as both availability and actual use, to testing services for the vulnerable populations they were intended to reach, particularly racial and ethnic minorities, people living in poverty and elderly individuals.

## Methods

We use data recorded at 20 community-testing sites managed by the NOHD between May 1 – June 23, 2020, or 52 effective days of testing. On average, 250 tests were administered each day. For each person tested, healthcare staff electronically recorded the following standardized information: site and date of test, patient’s race and ethnicity, date of birth, gender, exact address and test result. Exact addresses were taken from driver’s license or ID, and verbally confirmed as current address of residence.

Those addresses were then geocoded using the batch geocoding function in Google Earth Pro, which uses a more intuitive interpretation of even poorly or partially recorded addresses. This tool returned 97.9% of addresses correctly geocoded, they were then exported and reprojected in QGIS 3.10.8 A Coruña.

A total of 9721 people were tested at walk-up sites during the 52 days period, out of which 9521 addresses were properly geocoded (2.1% could not be matched). We excluded 171 observations where residence was more than 350 miles (450 km) away, for people who had recently moved to the city but could not provide a local address. A total of 9350 observations remained.

A separate layer geocoding the walk-up testing sites was also created and we used the “distance to nearest hub” and “distance matrix” functions to calculate the following variables [1]: distance between each person’s address and the nearest testing site they could potentially have visited to get tested [2], distance between each patient address and the testing site they actually did visit, and [3] difference between [1] and [2], which yielded “additional distance” covered to get tested. All distances were Euclidian, which is an acceptable alternative to network distance in a densely gridded city such as New Orleans [15].

T-tests were used to evaluate the statistical significance of differences between census tracts with and without a testing site nearby (*p*-value < 0.05). We then used logistic regression for binary outcome variables and multivariate linear regression models for continuous outcome variables to test whether gender, age, racial and ethnic identities, and test results were associated with visiting the nearest available testing sites and with traveling longer or shorter distances to obtain a test. We report significance levels at *p* < 0.01, *p* < 0.05 and *p* < 0.1.

A secondary dataset was obtained on the 9729 persons tested at drive-through sites (as opposed to walk-up) between March 20th and April 10th, out of which we were able to geocode 9515 addresses (error rate: 2.2%). These records however did not include reliable information on race and ethnicity; thus, it was only used to analyze differences in the percentage of persons tested who were Orleans parish residents at the two types of sites.

## Results

### Testing availability in low-income and minority neighborhoods

The shift from drive-through to walk-up sites increased the percentage of the persons tested who were residents of Orleans Parish from 45.8 to 78.5%, thus improving utilization by the intended beneficiaries. The drive-through locations were more likely to attract persons from the neighboring parishes (41.2%) or other parts of Louisiana (12.0%). (Table 1).

**Table 1:** Residence of persons tested at drive through and walk-up sites in Orleans parish, April – June 2020

Testing sites	Total	Orleans Parish	Jefferson – St Bernard Parish	Rest of Louisiana	Out of state
Drive through	9729	45.8%	41.2%	12.0%	0.9%
Walk-up	9712	78.5%	15.3%	2.4%	3.8%

**Fig. 1:**
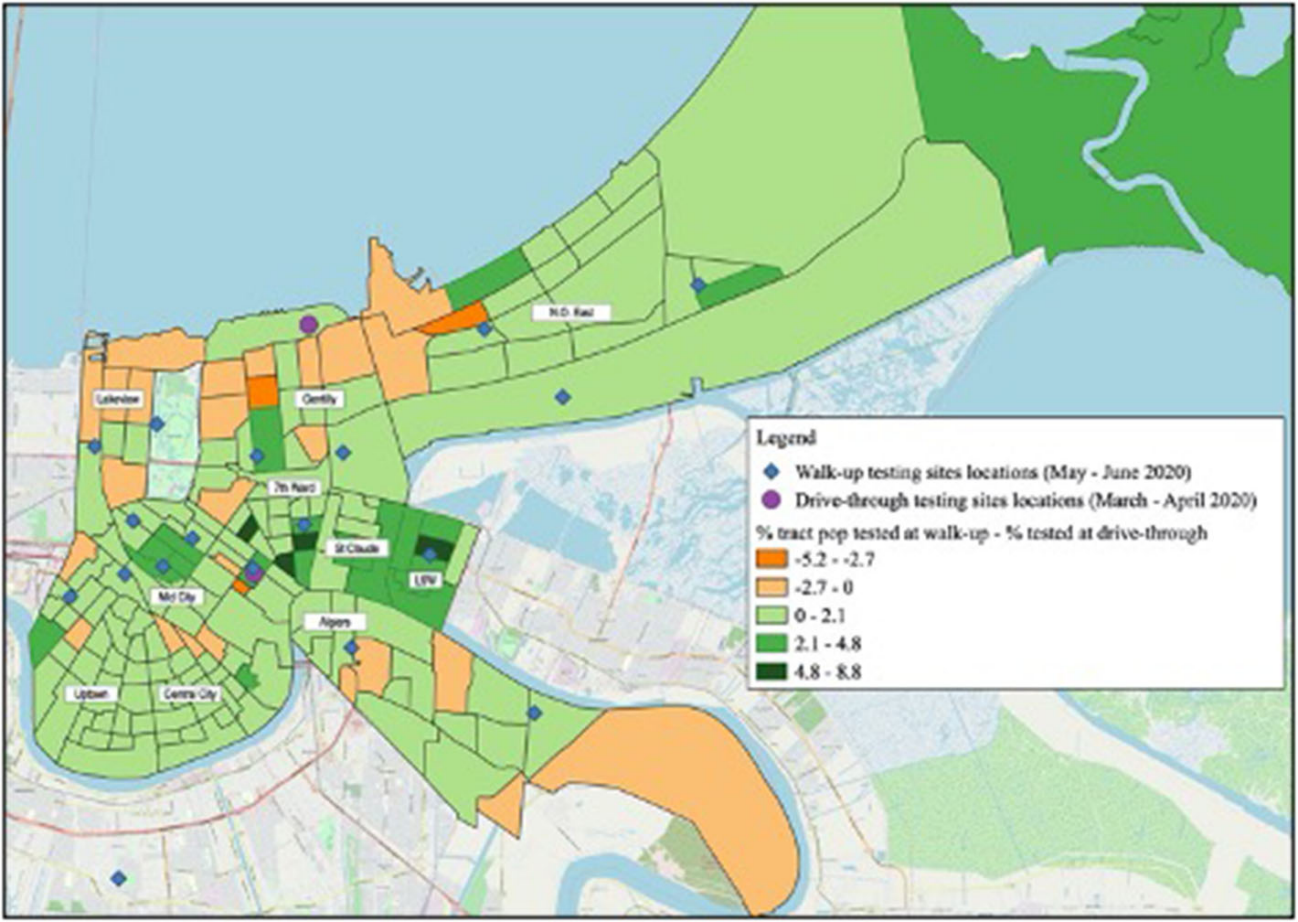
Difference between percentage of Orleans parish tract population tested at walk-up compared to drive-through sites

Changing from drive-through to walk-up sites also doubled the mean percentage of the population of each census getting tested. On average 1.2% of the population in each Orleans parish tract was tested at drive-through sites (median = 0.97%, maximum 8.3% minimum 0.0%, 3 tracts with less than 0.1% of the pop tested) against 2.2% at walk-up sites (median = 1.7% Maximum 10.5% Minimum 0.5%, no tract with less than 0.5% of the population tested). Figure 1 below shows the changes in percentage of each tract population tested at walk-up vs. drive-through sites.

The walk-up sites also partially succeeded in increasing availability of testing services in majority African American neighborhoods (e.g., the Lower Ninth Ward, St Claude or the 7th Ward, see Fig. 1.). Table 2 below describes the differences between census tracts with a testing site nearby (i.e., tracts either containing, or adjacent to a tract containing, a walk-up site) and those that did not. The demographic distribution for Orleans Parish is given for reference.

**Table 2:** Demographic of census tracts and their proximity to walk-in testing sites, ACS 2018

Demographics of the census tract	Orleans Parish	Tracts WITHOUT a walk-up site nearby	Tracts WITH a walk-up site nearby	*p*-value (t-test)
% Under federal poverty level	26.1	24.1	28.0	0.111
% White	36.9	44.3	29.9***	0.003
% African American or Black Hispanic	57.2	50.1	64.0***	0.006
% Non-Black Hispanic	5.7	5.5	5.8	0.661
% Asian	2.4	2.1	2.6	0.520
% Minorities	63.1	55.7	70.1***	0.003

*** *p* < 0.01, ** *p* < 0.05, * *p* < 0.1

Consistent with the objective of improving access to testing sites for minorities and vulnerable populations within Orleans Parish, walk-up sites were located in neighborhoods with significantly less white and more African American and minority populations. However, there were no significant differences in poverty levels of the census tracts that did or did not have a walk-up site nearby.

### Distance traveled by individuals to get tested

In addition to increased coverage, the analysis of distances actually travelled by individuals provided important insights into accessibility and utilization patterns. For all individuals, the mean distance to the closest testing site available during that period was 4.3 km (2.7 miles) with a median at 1.6 km (1 mile), whereas the mean distance to the site they actually used was 9.3 km (5.8 miles) with a median at median at 6.3 km (3.9 miles). Only 20.3% of all patients went to the nearest testing sites, and individuals on average covered an additional 5 km (3.1 miles) to get tested.

Using multivariate regression analysis, we tested the association between characteristics of the persons tested (test result, age, gender and ethnic group) and five dependent variables related to their testing experience: distance to the nearest testing site, whether the individual got tested at the nearest site, actual distance covered to get tested, additional distance traveled (beyond the nearest testing site), and result of the test. Key findings, shown in Table 3, were as follows.

**Table 3:** Multivariate regression analyses results

	(A)	(B)	(C)	(D)	(E)
	Distance to nearest site (Km)	Tested at nearest site (y/n)	Distance covered (Km)	Additional distance covered (Km)	Tested positive (y/n)
Covariates					
Distance to nearest site (Km)	–	−0.0014***	1.0227***	–	–
	–	(0.0002)	(0.0026)	–	–
Tested positive	–	0.0024	1.2443***	1.3080***	–
	–	(0.0264)	(0.3166)	(0.3178)	–
Male	1.1697***	−0.0024	0.2081**	0.2343**	0.0063**
	(0.3712)	(0.0079)	(0.0941)	(0.0944)	(0.0031)
African American or Black Hispanic	−1.1603**	0.1465***	−0.8358***	− 0.8623***	0.0012
	(0.4916)	(0.0104)	(0.1246)	(0.1251)	(0.0041)
Asian	−2.0179*	0.5299***	−3.1700***	−3.2152***	−0.0107
	(1.1722)	(0.0248)	(0.2971)	(0.2982)	(0.0097)
Hispanic white	1.0472	0.0300*	0.7451***	0.7579***	0.1733***
	(0.7742)	(0.0170)	(0.2037)	(0.2045)	(0.0064)
Native American	−4.0879	0.1273	−3.4612**	−3.5602**	0.0948*
	(5.9261)	(0.1253)	(1.5020)	(1.5079)	(0.0491)
Pacific Islander	−3.9385	− 0.0978	4.3046**	4.2158**	−0.0112
	(6.7180)	(0.1420)	(1.7024)	(1.7091)	(0.0557)
2 or more races	−1.7008	0.2377***	−1.5963***	−1.6351***	0.0012
	(1.8819)	(0.0398)	(0.4769)	(0.4788)	(0.0156)
Unknown race	−2.5330***	−0.0101	−1.6781***	−1.7360***	0.0044
	(0.5569)	(0.0118)	(0.1413)	(0.1417)	(0.0046)
Age < 15	−0.3027	0.0128	0.8140***	0.8059***	0.0178*
	(1.2176)	(0.0257)	(0.3086)	(0.3098)	(0.0101)
Age 15–19	− 0.2634	−0.0427	0.9214**	0.9139**	0.0237**
	(1.4246)	(0.0301)	(0.3611)	(0.3625)	(0.0118)
Age 20–24	3.1275***	−0.0244	0.5111*	0.5810**	0.0183*
	(1.1408)	(0.0241)	(0.2892)	(0.2903)	(0.0095)
Age 25–29	−0.0866	− 0.0168	0.2460	0.2436	0.0071
	(1.0255)	(0.0217)	(0.2599)	(0.2609)	(0.0085)
Age 30–34	−0.7911	−0.0020	0.1355	0.1186	−0.0167**
	(0.9896)	(0.0209)	(0.2508)	(0.2518)	(0.0082)
Age 35–39	−0.7586	0.0218	0.1209	0.1037	−0.0009
	(1.0157)	(0.0215)	(0.2574)	(0.2584)	(0.0084)
Age 45–49	−0.4260	0.0032	0.3175	0.3074	0.0051
	(1.1108)	(0.0235)	(0.2815)	(0.2826)	(0.0092)
Age 50–54	−0.3513	0.0565**	0.4611*	0.4538*	−0.0115
	(1.0585)	(0.0224)	(0.2682)	(0.2693)	(0.0088)
Age 55–59	0.2206	0.0873***	−0.0485	−0.0420	−0.0228***
	(1.0161)	(0.0215)	(0.2576)	(0.2586)	(0.0084)
Age 60–64	−0.4720	0.0871***	−0.1538	−0.1633	− 0.0191**
	(0.9668)	(0.0204)	(0.2451)	(0.2460)	(0.0080)
Age 65–69	−1.8589*	0.0986***	−0.0860	−0.1270	− 0.0202**
	(0.9593)	(0.0203)	(0.2432)	(0.2441)	(0.0080)
Age 70–74	−0.0584	0.1322***	−0.4275*	−0.4279*	− 0.0154*
	(1.0031)	(0.0212)	(0.2542)	(0.2552)	(0.0083)
Age > 74	−1.7704*	0.1496***	−0.4345*	−0.4732*	− 0.0244***
	(0.9956)	(0.0211)	(0.2524)	(0.2534)	(0.0083)
N	9350	9350	9350	9350	9350

*Notes*. Associations of covariates with distance to the nearest testing site are reported in column A and associations with a binary variable indicating whether the respondent got tested at the nearest testing site are shown in column B. Column C reports the associations of covariates with actual distance traveled to get tested. Column D includes the estimated associations of the covariates with additional distance traveled with respect to the nearest testing site. Finally, column E shows the estimated coefficients of each covariate on the likelihood of testing positive. All regressions were run as multivariate linear regression models. Reference categories were female, White, and age group 40–44

Standard errors in parentheses

*** *p* < 0.01, ** *p* < 0.05, * *p* < 0.1

Distance to nearest testing site was significantly shorter for both African Americans and Asians than for other groups (1.2 km (se = 0.49) and 2.0 km (se = 1.17) closer respectively), confirming earlier findings of increased testing availability in minority neighborhoods. Results suggests that increased availability may have translated into higher use: African Americans were 14.7% (se = 1.04) and Asians were 53.0% (se = 2.48) more likely to get tested at the nearest site, compared to Whites. Strongly significant associations also held for patients identifying with “two or more races,” which in New Orleans includes a large percentage of people with African American ancestry. Consequently, individuals from both groups actually covered much shorter distances to get tested: African Americans travelled about .5 miles (0.836 km, se = 0.125) and Asians about 2 miles (3.17 km se = 0.297) less than Whites to get tested.

By contrast, being Hispanic was not associated with increased proximity to and use of mobile sites and Hispanics traveled an additional .5 miles (0.745 km, se = 0.20) to get tested. This group is also the only one for which the likelihood of testing positive was significantly higher (+ 17.3%) than for Whites (*p*-value < 0.01).

As mobile sites were not located to serve specific age groups, there were no clear pattern of increased proximity of these sites to older age categories. However, being elderly was significantly and positively associated with testing at the nearest site, starting at age 50–54; these individuals were 5.65 (se = 2.24) percentage points more likely to get tested at the nearest site than the reference group of individuals 40–44. The strength of this association increased with age, with individuals 75 and older 14.96 (se = 2.11) percentage points more likely to get tested at the nearest site.

Finally, individuals who tested positive were significantly more likely to cover longer distances to get tested, on average 0.77 miles (1.24 km, se = 0.31) more than individuals who tested negative.

## Discussion

The analyses presented above provide programmatic insights validating the walk-up sites as a strategy to access to Covid-19 testing in vulnerable communities. In a city where limited private vehicle ownership reflects marked racial and economic inequities, local health officials quickly became aware of the limitations of the drive-through testing model. At the metropolitan level, and compared to drive-through sites, the walk-up sites succeeded in increasing testing for residents of Orleans Parish. The site selection for the walk-up sites also improved the availability of testing services in minority neighborhoods, particularly for predominantly African American census tracts.

When looking at individual behaviors, both African American and Asian patients were significantly more likely to use the nearest walk-up site than Whites, and overall covered a shorter distance to get tested. The same effect is observed for elderly populations, who were significantly less likely to get tested at sites farther from their home. The strength of this association increased linearly with age after 50: individuals above 74 years were those who traveled the least distance to get tested. This suggests that elderly individuals took advantage of the walk-up testing sites to avoid the previously documented [16] mobility barriers they face in accessing healthcare services located far from their homes.

By comparison, the Hispanic population appears not to have benefited from the walk-up sites as much as other racial and ethnic groups, as they were significantly more likely to cover additional distance to obtain a test. This is partially explained by the fact that most of the Hispanic minority in the Greater New Orleans Area resides in the suburbs of Kenner and Metairie in Jefferson Parish, and thus had to come to Orleans Parish to get tested at a walk-up site. Considering the significantly high positivity rate among Hispanics, this suggests a need for stronger testing coordination across administrative boundaries in large metropolises.

Finally, the longer distances covered by those who tested positive, after controlling for age and race, may indicate that individuals who perceive themselves at higher risk due to symptoms or positive contacts may be more willing to travel farther to receive a test.

Overall, these analyses suggest that community walk-up sites were successful in offering testing opportunities for at-risk and hard-to-reach populations within Orleans parish. With other conditions of costs and cultural competence being similar at sites across the city, proximity to home and familiar settings contributed to increasing access for vulnerable individuals. It is unclear however if walk-up sites supported better case management and follow-up care post-diagnosis. As populations facing barriers to testing also overlap with those likely to live in more crowded households, have poor job security and benefits in case of absence and suffer from limited access to medicalized care [4, 17, 18], they may not be able to heed isolation and symptom monitoring recommendations provided at the sites despite learning of their test result. In that perspective, it would be useful for future research to investigate whether the community sites settings and counselling strategies contributed to implement quarantine recommendations and contact tracing efforts more effectively than testing services offered at healthcare facilities.

The findings of this study must be considered in the light of several limitations. First, they were not produced out of an experimental or quasi-experimental design but rather as an evaluation of the outcomes related to the strategy implemented by NOHD and its partners. This has particular bearing as not all walk-up sites were equally available during the study period, and service statistics data only reflect populations who did take advantage of those sites but do not provide information on the existing need for testing in those populations. As testing behavior might be guided by perceived risk or external requirements (employment, travel), it was not guaranteed that the nearest walk-up site to any individual was operating at the time they decided to get tested. Second, positivity rate cannot be generalized to the whole population, as individuals self-selected for the service. Further, because of the structure of our data, we could not capture any mediator that may have been present on the pathway between significant explanatory variables and outcome variables such as income, insurance status, or employment type for individuals tested at walk-up sites. Third, the address recorded on the testing form was the patient’s residence and we could not estimate the proportion of individuals who received a test at a convenient community location that was not the nearest to their home (e.g., on their way to work, near relatives, etc.). Fourth, our sample includes more than nine thousand records, but encompasses 52 days of testing only; the observed patterns may not have held, had the period of the analysis been extended. Finally, while our primary dataset is fairly unique in recording exact addresses, race and ethnicity for individuals tested at walk-up sites, it is not exempt of data entry and errors in recording individual characteristics. The analysis conducted in this paper however demonstrate the importance of such a level of granularity in recording individual information to avoid blurring demographic disparities in Covid-19 exposure, infection and morbidity among vulnerable populations [19].

## Conclusions

Cities around the U. S are in the planning stages of vaccination campaigns that will only produce the required level of collective immunity if no viral reservoirs are ignored. In addition, immunization campaigns can only be equitable if racial, ethnic and economic minorities are covered proportionally to their vulnerability. Full population immunization through hospitals, primary care and pharmacy services is not realistic as the fault lines of access to formal care are likely to replay precisely for those groups who suffer the most from Covid-19 morbidity and mortality. Community-based strategies such as the walk-up sites presented in this article improve both coverage and access for hard-to-reach populations. With adequate technical (e.g., vaccine dose refrigeration) and messaging (e.g., addressing local reticence to immunization) adaptations, they could constitute a key complementary approach to facility sites.

## Data Availability

Datasets analyzed during the current study are not publicly available due to HIPAA restrictions. Anonymized data, with addresses aggregated at the block level, are available from the New Orleans Health Department and the corresponding author on reasonable request.
